# Healthcare Workers' Well-Being: A Systematic Review of Positive Psychology Interventions

**DOI:** 10.7759/cureus.34102

**Published:** 2023-01-23

**Authors:** Alexandra P Townsley, Jenny Li-Wang, Rajani Katta

**Affiliations:** 1 Psychology, Rice University, Houston, USA; 2 English, Rice University, Houston, USA; 3 Internal Medicine, Baylor College of Medicine, Houston, USA; 4 Dermatology, The University of Texas Health Science Center at Houston (UTHealth Houston), Houston, USA

**Keywords:** physician well-being, healthcare workers, mindfulness meditation, wellness and resilience, cognitive load, three good things, gratitude, mindfulness-based stress reduction, positive psychology

## Abstract

Given persistent occupational stressors and multiple challenges in the delivery of healthcare, there is an increased focus on the well-being of healthcare workers. Responding to these challenges will require a multipronged approach, focusing on system level, organization, and individual actions. Positive psychology interventions (PPIs) represent a promising area for individual action. This systematic review indicates that PPI, delivered via many methods, holds promise for improving the well-being of healthcare workers, although there is a clear need for additional randomized controlled trials utilizing defined and standardized outcome measures. In this review, the most commonly evaluated PPIs were mindfulness-based or gratitude-based interventions. These were delivered via different methods, with many administered in the workplace and commonly in the form of courses ranging from two days to eight weeks. Researchers documented measurable improvements in multiple studied outcomes, noting reductions in symptoms of depression, anxiety, burnout, and stress. Some interventions increased well-being, job and life satisfaction, self-compassion, relaxation, and resilience. Most studies emphasized that these are simple, accessible, low-cost interventions. Limitations included some nonrandomized or quasi-experimental designs, alongside generally small sample sizes and varying methods of intervention delivery. Another concern is the lack of standardized outcome assessments and long-term follow-up data. As almost all studies included were performed before the pandemic, further research will be required post-pandemic. Overall, however, PPI shows promise as one arm of a multipronged approach to improving the well-being of healthcare workers.

## Introduction and background

With a renewed focus on the well-being of healthcare workers in the face of increased occupational stressors [1], there is increased interest in the development of effective and easily implemented strategies that improve healthcare workers' well-being. The detrimental effects of the COVID-19 pandemic on the mental health and well-being of the healthcare workforce have been well-documented and extensively studied. Although elevated levels of depression, anxiety, and burnout among physicians and other healthcare workers are by no means a new phenomenon, the unprecedented conditions of the pandemic have greatly exacerbated this well-established phenomenon [2]. Heavy patient loads, long and irregular work hours, an unprecedented fall in employment early in the pandemic [2,3], and patient mistrust [4] have contributed to increased anxiety, depression, insomnia, and low self-efficacy [5]. A range of interventions will be needed to support healthcare workers' well-being in light of these stressors. A review of previously employed interventions may provide some guidance.

Responding to these challenges and improving the well-being of healthcare workers will require a multipronged approach, focusing on system-level, organizational, and individual actions. Promising interventions that may be employed by individual workers have been described in the positive psychology literature. Positive psychology is a field of study established by Seligman et al. in the early 2000s, with a focus on studying “positive emotions, positive character traits, and enabling institutions” [6]. Positive psychology interventions (PPIs) have been found to increase well-being and decrease depression from baseline in the general population with continued adherence, with effectiveness varying across interventions [6]. Multiple PPIs have been described; in this paper, we focus on those studied specifically in healthcare workers.

One frequently investigated PPI in the general population is mindfulness-based interventions (MBIs). Mindfulness in itself is a state of active awareness and attention directed at the present moment and includes nonjudgmentally attending to one’s current feelings and experiences [7]. Examples of MBIs include mindful movement programs, mindfulness meditation, and the body scan, in which awareness is focused on different areas of the body to progressively relax [7]. Another commonly studied PPI is the *Three Good Things* or *Three Blessings* intervention. In this exercise, participants write down three good things that happened in their day as well as the causes for them [8]. Multiple trials have also studied combinations of interventions, including PPI and other interventions that are based on positive psychology concepts and research.

The purpose of this study is to systematically review studies that evaluated PPI in healthcare workers to determine if PPIs help improve healthcare workers' well-being.

## Review

Methods

**Figure 1:**
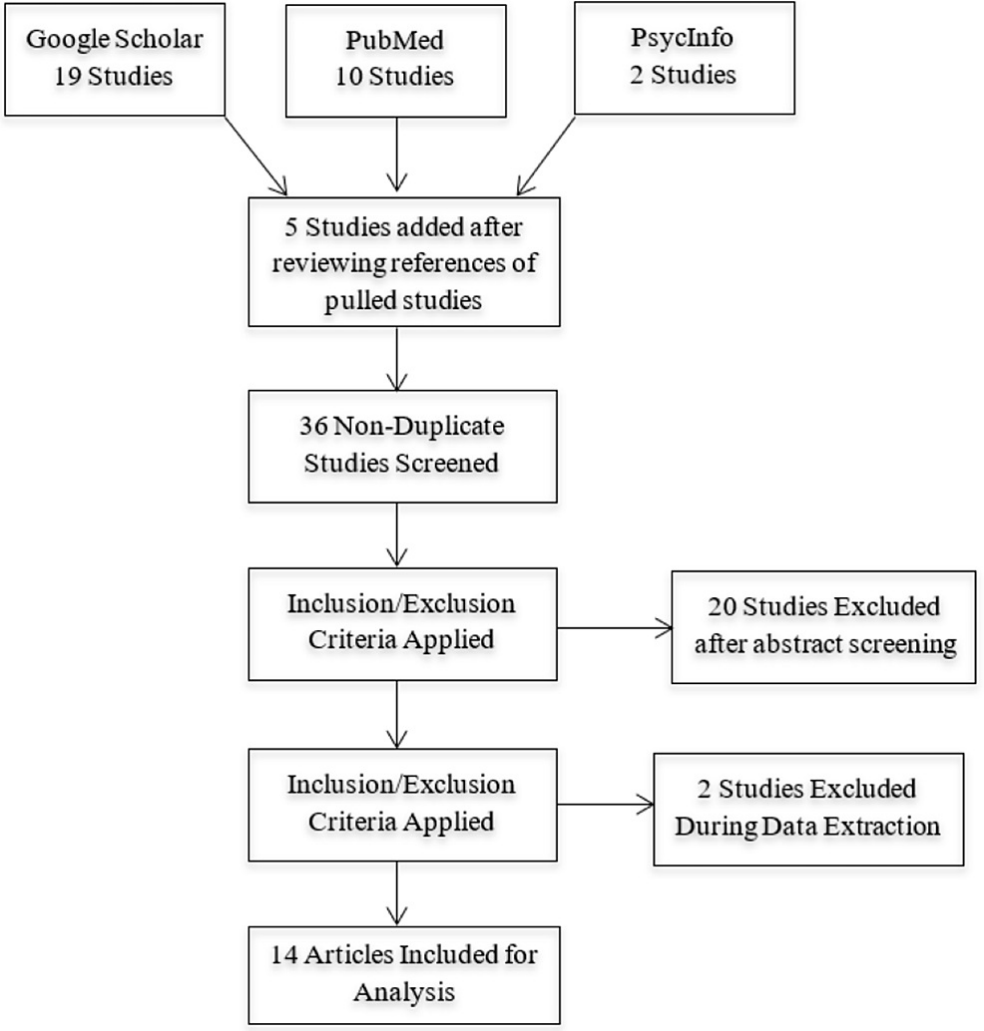
PRISMA diagram showing the study identification and selection process. Figure credits: Alexandra P. Townsley. PRISMA, Preferred Reporting Items for Systematic Reviews and Meta-Analysis

This study employed the Preferred Reporting Items for Systematic Reviews and Meta-Analysis (PRISMA) to organize and describe the findings of this systematic review (Figure 1). A literature search was performed from August to October 2022.

Search Strategy

A primary literature search was conducted using Google Scholar, PubMed, and PsycInfo databases. The search terms used were “positive psychology interventions,” “positive psychology in healthcare,” and “positive psychology in medicine.” All articles meeting the search criteria with full-text articles available were selected. A total of 36 articles were selected initially: 10 from PubMed, 2 from PsycInfo, and 19 from Google Scholar. Reviewing the references of included studies identified five additional articles.

Inclusion criteria were as follows: studies must be based on positive psychology concepts, written in English, and with full-text availability. The studies must employ a positive psychology-based intervention, with currently employed healthcare workers as subjects, and outcome measures must be defined, measured, and related to well-being. Studies that did not meet inclusion criteria included review articles and editorials, those with patients as subjects, and those measuring treatment outcomes, communication, or patient satisfaction as outcome measures. Of the initial 36 articles, 22 were excluded for not meeting inclusion criteria, resulting in 14 articles being analyzed and included in this study. Data from included studies included the study title, intervention and duration, number and occupation of participants, outcomes, and study design. The information extracted from the included articles is summarized in Table 1.

**Table 1:** Positive psychology interventions in HCWs. Adapted from [9-22]. RCT, randomized controlled trial; MBSR, mindfulness-based stress reduction; RN, registered nurse; 3GT, Three Good Things; PPT, positive psychology tool; CMA, certified medical assistant; HCW, healthcare worker

Paper	Authors	Study description	Intervention description	Subjects	Outcomes
A Brief Mindfulness-Based Stress Reduction Intervention for Nurses and Nurse Aides	Mackenzie et al. [9]	RCT	Four-week mindfulness intervention	30 nurses and aides	With respect to job-related personal accomplishment, intervention participants reported higher levels than controls both before and after the intervention. MBSR participants demonstrated reductions in exhaustion, whereas control participants’ scores increased somewhat. With respect to depersonalization, intervention participants showed relative stability over the two testing periods, whereas control participants’ scores increased significantly.
Mindfulness-Based Stress Reduction for Health Care Professionals: Results From a Randomized Trial	Shapiro et al. [10]	RCT	An eight-week MBSR program	38 physicians, nurses, social workers, physical therapists, and psychologists	Compared with controls, the intervention (MBSR) group demonstrated a significant mean reduction (27% versus 7%) in perceived stress and an increase in self-compassion (22% versus 3%). In the MBSR group, 88% of the participants improved their stress scores while 90% demonstrated increases in self-compassion. In addition, the MBSR condition demonstrated trends toward greater positive changes in all of the dependent variables examined. Compared with controls, intervention participants reported greater satisfaction with life (19% versus 0%), decreased job burnout (10% versus 4%), and decreased distress (23% versus 11%).
The Mindfulness-Based Stress Reduction Program (MBSR) Reduces Stress-Related Psychological Distress in Healthcare Professionals	Martín-Asuero and García-Banda [11]	Nonrandomized pre-post intervention study within-group design	An eight-week mindfulness-based stress reduction course involving 28 hours of classes	29 healthcare professionals	Results show a 35% reduction in distress, from percentile 75 to 45, combined with a 30% reduction in rumination and a 20% decrease in negative affect. These benefits lasted during the three months of the follow-up period.
A Mindfulness Course Decreases Burnout and Improves Well-Being Among Healthcare Providers	Goodman and Schorling [12]	Pre-post-follow-up within-subjects design	MBSR course that met for 2.5 hours a week for eight weeks plus a seven-hour retreat	93 physicians from multiple specialties, nurses, psychologists, and social workers	Burnout Inventory scores improved significantly for both physicians and other healthcare providers for the measures of emotional exhaustion, depersonalization, and personal accomplishment. Mental well-being also improved significantly. There were no significant changes in the physical health scores.
The Impact of an Innovative Mindfulness-Based Stress Reduction Program on the Health and Wellbeing of Nurses Employed in a Corporate Setting	Bazarko et al. [13]	Nonrandomized pre-post-intervention study within-group design	An MBSR program for eight weeks	36 nurses	Statistically significant improvements were observed on almost every measure, including self-kindness, common humanity, mindfulness, and overall self-compassion from baseline to postintervention, and were sustained four months later.
Improving Mental Health in Health Care Practitioners: Randomized Controlled Trial of a Gratitude Intervention	Cheng et al. [14]	Double-blind RCT	Participants in the intervention group wrote work-related gratitude and hassle diaries, respectively, twice a week for four consecutive weeks. A no-diary group served as control.	102 physicians, nurses, physiotherapists, and occupational therapists	The general pattern was a decline in stress and depressive symptoms over time, but the rate of decline became less pronounced as time progressed.
Preliminary Evaluation of a Brief Mindfulness-Based Stress Reduction Intervention for Mental Health Professionals	Dobie et al. [15]	Pre-post-follow-up within-subjects design	Daily 15-minute MBSR training over eight weeks interspersed with three 30-minute education sessions	Nine mental health workers: five nursing and four allied health (social work, occupational therapy, psychology) staff	Quantitative and qualitative participant feedback revealed a perceived reduction in psychological distress.
A Pilot Evaluation of a Mindful Self-Care and Resiliency (MSCR) Intervention for Nurses	Craigie et al. [16]	Pre-post-follow-up within-subjects design	Mindfulness-based self-care and resiliency intervention: one-day compassion fatigue prevention educational workshop, followed by weekly mindfulness training seminars over four weeks (12 hours total intervention time)	21 nurses	Significant improvements were observed for compassion, satisfaction, burnout, trait-negative effects, obsessive passion, and stress scores. At preintervention, 45% of the sample with high burnout scores was reduced to 15% postintervention. No significant changes were observed for general resilience, anxiety, or secondary traumatic stress.
Effect of Positive Psychological Intervention on Posttraumatic Growth Among Primary Healthcare Workers in China: A Preliminary Prospective Study	Xu et al. [17]	Nonrandomized pre-post-intervention study within-group design	A four-phase intervention designed based on positive psychology and Chinese culture: phase 1: baseline; phase 2: health education; phase 3: participants invited to ask questions about mental health and work difficulty followed by discussion; and phase 4: assessment	579 HCWs	Participants demonstrated improvement, using the Post-Traumatic Growth Inventory. The aspect of new possibilities improved the most with intervention. Women and nurses showed greater improvement than men and other professionals, respectively.
A Randomized Controlled Trial of Mindfulness to Reduce Stress and Burnout Among Intern Medical Practitioners	Ireland et al. [18]	RCT	A 10-week mindfulness intervention	44 intern doctors	Participants reported greater improvements in stress and burnout relative to controls.
Forty-Five Good Things: A Prospective Pilot Study of the Three Good Things Well-Being Intervention in the USA for Healthcare Worker Emotional Exhaustion, Depression, Work-Life Balance and Happiness	Sexton and Adair [19]	Nonrandomized survey design	Three good things intervention administered over 15 days	228 physicians, RNs, nurse managers/charge nurses, physician assistants/nurse practitioners, hospital aides, physical therapists, occupational therapists, pharmacists, respiratory therapists, technologists/technicians, administrative support, other managers, and students	3GT participants exhibited significant improvements in emotional exhaustion, depression symptoms, and happiness at one month, six months, and 12 months.
Enhancing Caregiver Resilience: Courses with Positive Psychology Tools Promote Durable Improvements in Healthcare Worker Burnout	Masoud et al. [20]	Nonrandomized survey design	One- or two-day resiliency course: Courses included didactics on burnout prevalence, strategies for coping and improving well-being, along with evidence-based PPTs used during and after the course.	1,396 nurses, physicians, pharmacists, clinical support (CMA, nursing aide, etc.), clinical social workers, physical/speech/occupational therapists, nutritionists, administrative support, and other health system employees	Higher baseline burnout and PPT use predicted the greatest improvements in HCW burnout. Participants of the two-day course exhibited significant improvements in burnout up to one month later; this group also reported higher baseline burnout.
An Evaluation of a Positive Psychological Intervention to Reduce Burnout Among Nurses	Luo et al. [21]	Quasi-experimental research design involving a study group and a control group	The 3GT intervention implemented using WeChat communication tool for six months	41 nurses	Nurses recording 3GT on average twice a week returned the lowest score of exhaustion.
The Effectiveness of an Online Positive Psychology Intervention among Healthcare Professionals with Depression, Anxiety or Stress Symptoms and Burnout	Alexiou et al. [22]	RCT	3GT with causal explanations for one week; five acts of kindness in a week; imagining the best possible self over weeks (different areas of life each day)	30 nurses, psychologists, social workers, and physiotherapists	The intervention group experienced statistically significant decreases in depression, anxiety, stress, and emotional exhaustion scores, as well as increases in satisfaction with life, compared to the control group, which reported no changes.

Discussion

This systematic review indicates that PPIs, delivered via a number of methods, hold promise for improving the well-being of healthcare workers, although there is a clear need for additional RCTs utilizing clear and standardized outcome measures.

PPIs, evaluated in multiple populations, have demonstrated measurable reductions in stress, anxiety, and burnout as well as improvements in job satisfaction and subjective well-being. These interventions have also produced physical benefits, including decreased inflammatory biomarkers and cortisol levels, as well as documented cognitive benefits, including increased working memory and decreased distractibility [23].

These benefits are of particular relevance to healthcare workers. The occupational challenges inherent in healthcare result in significant cognitive, emotional, and physical demands. Even under normal working conditions, physicians and other healthcare workers daily encounter stressful situations and intense cognitive and emotional demands. Cognitive demands include, among many others, the need to make critical healthcare decisions as well as the need to focus attention in the face of frequent distractions and interruptions. The emotional demands are significant as well, including caring for and communicating with distressed patients as well as self-regulation and emotion work in taxing situations [8].

These challenges were intensified during the COVID-19 pandemic, and although the pandemic has begun to wane, the mental health effects persist. Studies have consistently shown an increase in anxiety, depression, and stress experienced by healthcare workers throughout the pandemic. One study found that 98.5% of physicians experienced moderate-to-severe stress levels during the pandemic, with 90.5% reporting varying levels of anxiety and 94% reporting mild-to-severe depression [24].

Under these conditions, the development and evaluation of interventions focused on improving the well-being of healthcare workers are of high priority. Improving physicians' well-being requires a multipronged approach that incorporates system-level, organizational, and individual actions [1]. Interventions based on positive psychology principles may be useful at the individual level and offer a potentially low-cost, safe, and effective tool when used alongside systemic and organizational improvements.

The positive psychology literature has established the efficacy of PPI in the general population via multiple research studies, including multiple randomized controlled trials (RCTs) [6]. Most of these PPIs were designed specifically to be completed easily at home to increase one’s sense of subjective well-being. There are seven basic categories of PPI: savoring, gratitude, kindness, empathy, optimism, strengths, and meaning. Savoring is based on the idea that if one directs their focus and attention to a positive event, one can prolong the positive emotions that result from that intervention; mindfulness-based interventions often fall into this category [25]. Gratitude interventions are designed to focus attention on the people and things that create positive events and feelings in one’s life. Interventions based on empathy seek to strengthen social connections, which have been shown as essential for happiness. Optimism activities emphasize thinking about the future and creating positive expectations. Activities based on strengths encourage one to identify character strengths and then use them in new ways. Finally, meaning-based interventions are focused on understanding and engaging with one’s meaning in life [6].

In this literature review, the most commonly evaluated PPI in healthcare workers was based on mindfulness or gratitude. Of the 14 studies, 8 were mindfulness-based, with a focus on either stress reduction or self-care. Mindfulness-based interventions have been studied extensively in different populations. These may be administered via different methods and have demonstrated effectiveness in multiple RCTs. One RCT found that an eight-week mindfulness-based stress reduction (MBSR) intervention was well-tolerated and of comparable effectiveness as the medication escitalopram in the treatment of generalized anxiety disorder [26].

Interventions in this study ranged from MBSR to mindful self-care interventions and demonstrated diverse modes of administration. Most were administered in the workplace in the form of courses ranging from two days to eight weeks. Two trials designed a combined intervention focusing on resilience and mindfulness. These interventions sought to increase well-being by teaching coping strategies for stressors and adversity (resilience), along with mindfulness. Another study combined mindfulness education with general positive psychology education, while one study used a resilience-based intervention.

Mindfulness-based interventions have grown in popularity as research has demonstrated benefits. Healthcare workers in qualitative studies have described benefits for themselves, their colleagues, and their patients, such as nurses who reported improvements in coping with workplace stress and developing feelings of inner calm [27].

In the studies reviewed here, researchers documented measurable improvements among a host of studied outcomes, including stress, burnout, exhaustion, depression, anxiety, and others. Research continues into potential mechanisms of action for these benefits. Some may be mediated via physiological mechanisms. Breathwork, included in many mindfulness meditation practices, includes slow breathing practices. This practice increases links between the parasympathetic and central nervous systems, which produces higher order cognitive control and mental flexibility [28]. During times of physiological stress response activation, therefore, the neural centers responsible for interpreting and reducing stress responses are also activated, resulting in stress reduction.

Mindfulness has also demonstrated anti-inflammatory benefits. One negative effect of stress is the upregulation of inflammatory biomarkers. Mindfulness meditation may counteract the negative inflammatory effects of stress by changing gene expression over time to downregulate inflammatory proteins [28]. Mindfulness-based interventions also exert effects on stress responses via neural pathways in the brain. Structural MRIs have demonstrated that these practices are associated with diminished activity in the amygdala in response to stress-inducing stimuli while in “mindful states as well as in a resting state” [29]. In other words, while experiencing stress, the emotion and fear center of the brain is less aroused in those who practice mindfulness than in those who do not.

Mindfulness may also prove beneficial via impacts on cognitive load. Any task performed by a healthcare worker inherently involves a degree of cognitive resources and effort, known as intrinsic cognitive load [30]. Extraneous cognitive load is not inherent to the task but may be impacted by inefficiencies in the system. Mindfulness can reduce cognitive load by increasing working memory capacity while simultaneously reducing the occurrence of distracting thoughts [23]. Even brief mindfulness meditation sessions have been shown to improve executive attention [31]. Reducing the overall cognitive load may prove beneficial to the well-being of healthcare workers [32].

Four of the interventions in this review were gratitude-based. Some of these interventions were traditional “three good things” interventions. In these interventions, participants are asked to write about three good things that they are grateful for each day, along with causal explanations of how they brought about those things or events. Other gratitude interventions were focused on keeping gratitude journals or diaries. Gratitude is generally thought to be effective for increasing well-being and reducing symptoms of anxiety and depression owing to a more positive view of the self and a focus on the positive emotions and events created by gratitude [33]. Gratitude interventions that prompt the expression of gratitude to others may also increase well-being through strengthened relationships [33].

In this review, overall, PPIs were effective at reducing symptoms of depression, anxiety, burnout, and stress while increasing well-being, job, and life satisfaction, self-compassion, relaxation, and resilience. Most studies emphasized that these PPI are simple, accessible, low-cost interventions that can be employed either at home or in the workplace. Therefore, they could potentially be easily replicated and deployed in other institutions.

Despite these promising results, several questions and limitations remain. Several studies used nonrandomized or quasi-experimental designs rather than the gold-standard RCT. The RCTs are limited by generally small sample sizes, as well as varying methods of intervention delivery. In addition, of the 14 studies in this review, only three provided information on participants' dropout rates, and reasons for dropout were not addressed. In these three studies, dropout rates were 0% [15], 12% [13], and 44% [10]. Another concern is the lack of standardized outcome assessments, limiting the comparison of the effectiveness of different administration methods. Additionally, long-term follow-up data is lacking, with the longest follow-up period here at three months. Long-term longitudinal data will be required to determine the duration of benefits, as well as to evaluate optimal intervals of intervention delivery. Another limitation of this systematic review is that the studies identified here, with one exception, all administered PPI before the onset of the pandemic. Publication bias should also be considered; although all studies in this review demonstrated positive outcomes, studies with negative outcomes may be less likely to be published.

In terms of future directions, high-quality RCTs that utilize clear and standardized outcome measures are needed to determine best practices for designing and delivering effective interventions. The development of standardized outcome measures should be a priority, and special focus should be paid to how healthcare workers can implement such practices in the face of challenging schedules. Future trials should also identify best practices for identifying individuals who are interested and who may benefit as well as closely monitoring rates and reasons for participants' dropout. Another question that remains is the effectiveness and feasibility of interventions implemented in the workplace setting versus interventions employed exclusively at home. Finally, another area for future study will be the assessment of the efficacy of PPI administered post-pandemic.

## Conclusions

In this systematic review of studies investigating the use of PPIs in healthcare workers, promising results were noted. Despite the current limitations, the studies reviewed here show promise as one arm of a multipronged approach to improving the well-being of healthcare workers. More research is needed into standardizing outcome measures and comparing different modes and methods of delivering these interventions.
